# CXCR4 Mediates Enhanced Cell Migration in *CALM-AF10* Leukemia

**DOI:** 10.3389/fonc.2021.708915

**Published:** 2022-01-05

**Authors:** Shelby A. Fertal, Sayyed K. Zaidi, Janet L. Stein, Gary S. Stein, Jessica L. Heath

**Affiliations:** ^1^ Department of Pediatrics, Larner College of Medicine, University of Vermont, Burlington, VT, United States; ^2^ Department of Biochemistry, Larner College of Medicine, University of Vermont, Burlington, VT, United States; ^3^ University of Vermont Cancer Center, Burlington, VT, United States

**Keywords:** CXCR4, CXCL12, leukemia, migration, adhesion, CALM-AF10 leukemia

## Abstract

Leukemia transformed by the *CALM-AF10* chromosomal translocation is characterized by a high incidence of extramedullary disease, central nervous system (CNS) relapse, and a poor prognosis. Invasion of the extramedullary compartment and CNS requires leukemia cell migration out of the marrow and adherence to the cells of the local tissue. Cell adhesion and migration are increasingly recognized as contributors to leukemia development and therapeutic response. These processes are mediated by a variety of cytokines, chemokines, and their receptors, forming networks of both secreted and cell surface factors. The cytokines and cytokine receptors that play key roles in *CALM-AF10* driven leukemia are unknown. We find high cell surface expression of the cytokine receptor CXCR4 on leukemia cells expressing the CALM-AF10 oncogenic protein, contributing to the migratory nature of this leukemia. Our discovery of altered cytokine receptor expression and function provides valuable insight into the propagation and persistence of *CALM-AF10* driven leukemia.

## 1 Introduction

The *t(10;11)(p13;q14-21)* chromosomal translocation results in the fusion of *CALM* (encoding the gene for the clathrin assembly lymphoid myeloid protein, also known as phosphatidylinositol-CALM (PICALM)) to *MLLT10* (also known as AF10) and is a recurrent genetic abnormality in acute lymphoid and myeloid leukemia. Nearly all of each individual gene is retained in this translocation. The endogenous CALM protein is primarily involved in intracellular trafficking. It associates with AP2 and clathrin, and functions to stabilize the clathrin-coated endocytic pit, allowing for efficient cellular uptake of growth factors and other receptor-bound molecules (1). Haploinsufficiency of CALM, as is found in cells expressing CALM-AF10, results in decreased endocytic efficiency (2). It has been reported that CALM contains a nuclear export signal (NES), allowing interaction with the CRM1/XPO1 nuclear export receptor. The NES is retained in the CALM-AF10 fusion construct, where it facilitates nucleocytoplasmic shuttling, and is necessary for leukemogenesis (3). AF10 is a transcription factor, binding to DNA through its leucine zipper domain, which is necessary for leukemogenesis (4–6). AF10 binds Dot1L, the only known H3K79 methyltransferase, and recruits Dot1L to its target genes, including the *HOXA* gene cluster (7). Upregulation of *HOXA* genes in *CALM-AF10* leukemias is a driving factor in *CALM-AF10* mediated leukemogenesis (8, 9).

The *CALM-AF10* translocation was first identified in a patient with histiocytic sarcoma but is more commonly seen in T acute lymphoblastic leukemia (T-ALL), where it makes up about 15% of pediatric and adult cases (10–12). *CALM-AF10* translocated T-ALL is characterized by the presence of extramedullary disease, CNS relapse, and poor response to therapy (12). This translocation is also seen in acute myeloid leukemia (AML), where it is associated with bulky hepatosplenomegaly, mediastinal disease, and central nervous system (CNS) leukemia (13).

Extramedullary and CNS leukemia development relies on migration of the leukemia cell out of the bone marrow compartment, and adhesion of the leukemia cells to cells of the target organ (CNS, liver, spleen, or lymph nodes). Multiple proteins have been implicated in this process, including several members of the integrin and chemokine families (14–16). More recently, interactions between leukemia cells and components of the bone marrow microenvironment have been shown to be critical for leukemic cell survival as well as resistance to chemotherapy [reviewed in (17)]. Interactions of leukemic cells with their microenvironment are mediated both by direct cellular contact and via soluble factors. One such factor is C-X-C motif chemokine receptor 4 (CXCR4), a cell surface G-protein coupled chemokine receptor that is bound and activated by C-X-C motif chemokine ligand 12 (CXCL12). CXCL12 can also enter the vascular system, and its interaction with CXCR4+ circulating hematopoietic stem cells (HSCs) is vital for HSC homing to the bone marrow compartment (18). The CXCR4/CXCL12 interaction plays a role in malignant hematopoiesis as well. Binding of CXCL12 to CXCR4 on leukemia cells activates multiple proliferative and survival pathways (reviewed in (19)), and high CXCR4 expression on leukemic blasts has been identified as a poor prognostic factor in both ALL and AML (20, 21). Finally, increased CXCR4 expression predicts the development of extramedullary disease in pediatric ALL (22).

We report an increase in cell surface expression of CXCR4, and a concomitant increase in the migratory behavior of cells transformed by *CALM-AF10*. Using both human and mouse cell line models, we examine the relationship between CXCR4 expression and phenotype in *CALM-AF10* transformed leukemias. Surprisingly, we do not find synergistic effect of CXCR4 inhibition with traditional chemotherapy in these leukemias, strongly suggesting caution in the broad use of these inhibitors, and a more tailored approach to this targeted therapy.

## 2 Methods

### 2.1 Cell Culture

Human leukemia cell lines were cultured in RPMI 1640 (Fisher Scientific, Waltham, MA, USA) supplemented with 100 units/mL penicillin, and 100ug/mL streptomycin. U937 and Fujioka cells were additionally supplemented with 10% fetal bovine serum (Atlanta, USA) and 2mM L-glutamine. Kasumi-1 cells were additionally supplemented with 20% fetal bovine serum and MEM non-essential amino acids. Human mesenchymal stem cells (hMSC) were cultured in α-MEM (ThermoFisher Scientific, Waltham, MA, USA) media supplemented with 16.5% FBS, 2mM L-glutamine, 100 units/mL penicillin, and 100ug/mL streptomycin. Murine leukemia cell lines were derived from cells isolated from the marrow of leukemic mice (a generous gift from Catherine Lavau, Duke University, North Carolina). *Hoxa9-Meis1* and *CALM-AF10* transformed cells were cultured in RPMI 1640 with 20% fetal bovine serum (Atlanta, USA), 100units/mL of penicillin and streptomycin, and 10ng/mL mouse recombinant IL-3 (Stem Cell Technologies). Murine embryonic fibroblasts (MEFs) were generated from *Picalm^fit1-5R^* E14 embryos, immortalized with SV40, as previously described (2). Plat-E cells were transiently transfected with the MSCV-IRES-GFP encoding *CALM-AF10* (3), or the MSCV-IRES-GFP empty control vector using calcium phosphate. MEFs were then retrovirally infected with these constructs by co-culture with filtered Plat-E supernatant. Infection efficiency was measured by flow cytometry at the time of cell line genesis, and intermittently confirmed using fluorescent microscopy and western blot analysis. All cell lines were incubated at 37°C with 5 percent CO_2_. All human cell lines and were validated continually by assessment of morphology and annually by short tandem repeat (STR) analysis. Murine cell lines were validated continually by assessment of morphology in cell culture and periodically by western blot assessment of the presence or absence of the CALM-AF10 fusion protein. In addition, all cells were confirmed negative for Mycoplasma infection with the Mycoalert detection kit (Lonza, Basel, CH) annually, or if any morphologic or growth changes were identified.

### 2.2 Immunofluorescence

Samples of 5x10^5^ cells per condition (CXCR4 stained and negative control) for human and murine cell lines were spun down at 1500 rpm for 5 minutes at room temperature, washed twice with PBS and fixed with 3.7% formaldehyde while rotating for 10 minutes. Fixed cells were washed twice with PBS, and resulting pellets were resuspended in 200uL of sterile filtered PBS per condition and transferred onto slides using the Shandon Cytospin 4 (ThermoFisher Scientific, Waltham, MA, USA). Cells were not permeabilized prior to staining, in order to specifically evaluate presence of CXCR4 at the plasma membrane. Slides were air dried for 5 minutes, washed in PBS with 0.5% BSA (PBSA), and either incubated in primary antibody against CXCR4, diluted 1:100 in PBSA (Abcam, Cambridge, UK), or PBSA for 1hr at 37°C in the dark. Samples were washed once in PBSA and incubated in secondary antibody anti-rabbit IgG Alexa Fluor 488, diluted 1:800 (ThermoFisher Scientific, Waltham, MA, USA), for 1hr at 37°C in the dark. Samples were washed in PBSA and stained with 0.5ug/mL DAPI for 5 minutes in the dark and washed once with PBSA. Slides were imaged using Zeiss Imager.Z2.

### 2.3 Western Blot

Total cellular protein was extracted from 1 x 10^6^ cells by boiling in 70uL of 1X protein loading buffer for 15 minutes. Protein samples were resolved in a 10% SDS gel at 140 V for 1.15hr, and the resolved proteins were transferred to a nitrocellulose membrane at 100 V for 2 hr. Membranes were blocked in 5% milk in Tris-Buffered Saline (TBS) for 1hr, then incubated overnight at 4°C in primary antibody against CXCR4 (Abcam Cambridge, UK) or CALM (Sigma, St. Louis, MI, USA), diluted 1:1000 in TBS containing 0.1% TWEEN. Following primary antibody incubation, membranes were washed 3x in TBST for 5 minutes and incubated with HRP-conjugated anti-rabbit antibodies (1:10,000) for 1 hour. After washing as above, membranes were developed with BioRad Molecular Imager ChemDoc XRS+ system using a Clarity Western ECL substrate kit (BioRad, Hercules, CA, USA). To confirm equal loading of proteins across the experimental conditions, membranes were subsequently incubated with a primary antibody against β-actin at 1:1,000 (Cell Signaling Technologies, Danvers, MA, USA), developed, and imaged as described above.

For co-culture experiments, 1.5x 10^5^/mL hMSC cells were plated on 10cm plates and incubated at 37°C with 5% CO_2_ for 24hrs. 4.5x 10^5^/mL U937 cells were added to previously cultured hMSCs and incubated at 37°C with 5% CO_2_ for 24hrs. Following the 24hr incubation, co-cultured cells were exposed to 1uM of AMD3465, a CXCR4 inhibitor (Selleck Chemicals, Houston, TX, USA), for 4hrs. Suspension U937 cells were harvested at 1x 10^6^/70uL of 1X loading buffer and boiled for 15 minutes. Samples were loaded on 15% SDS gel, run at 140 V for 3.36hr, and transferred to a nitrocellulose membranes at 100 V for 2 hr. Five percent BSA in Tris-Buffered Saline (TBS) was used to block membranes for 1hr. Membranes were incubated in primary antibody solutions with TBS containing 0.1% TWEEN for p44/42 (ERK) at 1:1,000 (Cell Signaling Technologies, Danvers, MA, USA) and phospho-p44/42 (phospho-ERK) at 1:2000 (Cell Signaling Technologies, Danvers, MA, USA) overnight at 4°C. Membranes were developed and imaged as stated above, and subsequently incubated in β-actin as described above.

All western blots are done in duplicate with one representative experiment shown.

### 2.4 Real-Time qPCR

Cells (1x 10^6^) were harvested in 300uL of Trizol reagent and RNA was extracted using Directzol RNA MiniPrep Plus kit (Zymo Research, Irvine, CA, USA) and quantified using the Thermo Scientific Nanodrop 2000. cDNA was synthesized using SuperScript III Reverse Transcriptase kit (Fisher Scientific, Waltham, MA, USA). Real-time PCR was performed on a ViiA 7 Real-time PCR with the ITaq Universal SYBR Green Supermix and probes for: human GAPDH forward (5’ ACCCACTCCTCCACCTTTGAC 3’), reverse (5’ TGTTGCTGTAGCCAAATTCGTT 3’); CXCR4 forward (5’ CACTTCAGATAACTACACCG 3’), and reverse (5’ ATCCAGACGCCAACATAGAC 3’); CXCL12 forward (5’ GGACTTTCCGCTAGACCCAC 3’), and reverse (5’ GTCCTCATGGTTAAGGCCCC 3’).

### 2.5 Scratch Assay

One million MEFs were plated in a 6 well tissue culture plate 24 hours prior to the experiment, allowing growth to confluence. At hour 24, a single scratch was made using a 200uL pipette tip. The media was aspirated off, each well was gently rinsed with PBS, and 2mL fresh media slowly added. Cells were photographed at hours 0, 4, and 8. Given the short time course of this experiment, anti-mitotic agents were not used. Photographs were processed by ImageJ to measure the area of the scratch at each time point.

### 2.5 Transwell Migration Assay

Corning transwell plates (8µm pore size, Fisher Scientific, Waltham, MA, USA) were used to assess migration. CXCL12 (R&D Systems, Minneapolis, MA, USA), or control (PBS), was added at 100ng/uL to 1.5x 10^5^ Kasumi-1 and U937 cells and incubated for 24hrs at 37°C with 5% CO_2_. After 24hrs, inserts were removed, each well was agitated *via* pipette, and cells were incubated for 30 minutes prior to imaging. Three images were taken of each corresponding well and representative areas of each image were analyzed *via* ImageJ (23). Two independent investigators performed each analysis to assure reproducibility. The above was repeated in U937 cells following a 4hr incubation in 0.25uM, 0.5uM, or 1uM AMD3465 (Selleck Chemicals, Houston, TX, USA) or control (water). Following a 4hr, 8hr, and 24hr incubation in transwell plates, images were taken and analyzed as detailed above.

### 2.6 Cytotoxicity Assays

#### 2.6.1 Flow Cytometry

Human MSC cells were plated at 1x 10^5^ cells/mL per well of a 6-well plate. Cells were cultured for 8hrs, then 3x 10^5^/mL U937 cells were added to the hMSCs culture. Co-cultures were maintained for 16hrs at 37°C with 5% CO_2_. Twenty-four hours from initial hMSC plating, Doxorubicin (1uM) and AMD3465 (1uM) (Selleck Chemicals, Houston, TX, USA) were added to co-cultured cells and allowed to incubate 37°C with 5% CO_2_ for 24hrs. Suspension cells were removed from wells and combined with corresponding adherent cells trypsinized with 0.25% trypsin. Samples were washed once in phenol free RPMI 1640 media. Non-specific binding was blocked with normal mouse IgG 1:200 (Santa Cruz Biotechnology, Dallas, TX, USA) in 1% BSA (50uL per 1x 10^6^ cells) for 30 minutes in the dark at 4°C. Primary antibody APC-conjugated mouse, anti-human CD45 (BD Pharmingen, San Diego, CA, USA) was added to cell solutions at 1:10, and incubated on ice for 30 minutes in the dark. Cells were washed once with phenol free RPMI 1640 and resuspended in 1uM DAPI solution. Samples were run on BD LSRII and analyzed using FlowJo software.

#### 2.6.2 BrdU Assay

Cells were plated at 5x10^5^ cells/mL media and treated with one of the following conditions: vehicle control (sterile water), 1uM AMD3465, 2uM AMD3465, 2.5uM cytarabine, 100nM cytarabine, 1uM AMD3465 + 2.5uM cytarabine, 1uM AMD3465 followed four hours later by 2.5uM cytarabine, 1uM AMD3465 + 100nM cytarabine, 1uM AMD3465 followed four hours later by 100nM cytarabine, 2uM AMD3465 + 100nM cytarabine, and 2uM AMD3465 followed four hours later by 100nM cytarabine. Treated cells were incubated at 37 degrees with 5% CO2 for 48 hours and were then analyzed by BrdU staining (Cell Signaling Technology, Danvers, MA, USA). Absorbance was read at 450nM on a Perkin Elmer multimode plate reader.

### 2.7 Statistical Analysis

All data are expressed as the mean ± Standard Error of the Mean (SEM), unless otherwise noted. Differences between two experimental groups were analyzed by unpaired t-test. Ordinary one-way ANOVA was used to compare differences between multiple groups. A p-value ≤ 0.05 was considered significant.

## 3 Results

### 3.1 Enhanced Cell Migration in CALM-AF10 Transformed Cells

**Figure 1:**
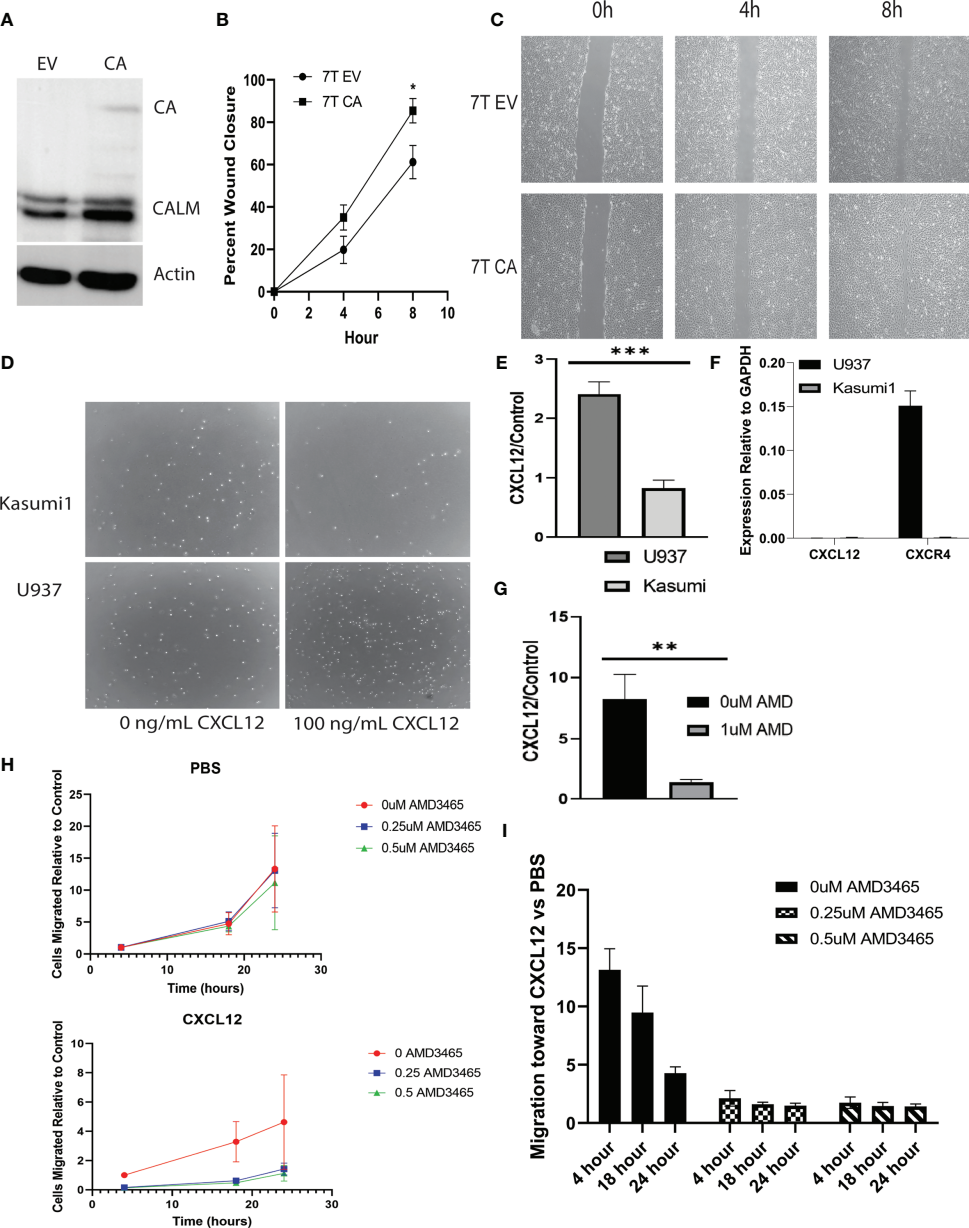
*CALM-AF10* leukemia cells show enhanced migration, influenced by CXCL12. **(A)** A Western blot of MEFs transformed by empty vector (EV) or CALM-AF10 (CA) confirms the presence of the CA oncoprotein. **(B)** Accelerated wound closure in murine embryonic fibroblasts (MEFs) transformed by *CALM-AF10*. **(C)** Representative images from one replicate of scratch assay demonstrate more rapid wound closure in *CALM-AF10* transduced MEFs. **(D)** Representative images from one transwell assay are shown. **(E)** The impact of CXCL12 as a stimulant for cell migration is examined in U937 and Kasumi cells. **(F)** qPCR of CXCL12 and CXCR4 in U937 and Kasumi1. **(G)** The impact of the CXCR4 inhibitor AMD3465 on the migration of U937 cells is evaluated. **(H)** Dose and time response curves of U937 cells exposed to AMD3465 with and without CXCL12 stimulation reveal migration U937 cells over time (normalized to the 0 AMD3465 condition). **(I)** Dose and time response experiments in U937 cells using AMD3465 in the present or absence of CXCL12 as a chemoattractant. *p < 0.05; **p < 0.01; ***p < 0.001.

Murine embryonic fibroblasts (MEFs) were stably infected with *CALM-AF10* or an empty vector (**Figure 1A**), and scratch assays were performed to assess the impact of the CALM-AF10 fusion oncoprotein on cell migration. Cells were plated the day prior to achieve confluence at the time of scratch. Closure of the wound was examined at four and eight hours post scratch infliction, and percent wound closure calculated. The *CALM-AF10* transformed MEFs showed a 35 percent wound closure at 4 hours, compared to 19% closure in the empty vector cells (p<0.05). This difference was magnified at the 8-hour time point, with CALM-AF10 transformed cells showing a 85% closure, compared with 61% in the empty vector cells (p=0.04) (**Figures 1B, C**).

We then examined the migratory behavior of human leukemia cell lines and sought to identify cytokines that could be involved in the migration of *CALM-AF10* leukemias. Because CXCL12 is the primary ligand for the CXCR4 receptor, we investigated the migratory behavior of *CALM-AF10* translocated leukemias when treated with CXCL12. We found that CALM-AF10 translocated U937 cells demonstrated an increase in cell migration upon stimulation with CXCL12, whereas the CALM-AF10 negative Kasumi-1 cells did not have a significant change in cell migration with the addition of CXCL12. U937 cells exhibited a nearly 3-fold increase in migration compared to Kasumi-1 cells when treated with 100ng/mL CXCL12 over a 24hr period (**Figures 1D, E**, n=3, p< 0.001). CXCL12 is measured in U937 and Kasumi1 cells by qPCR and reveals that CXCL12 is not expressed in either cell line at any significant level (**Figure 1F**: CXCR4 expression is shown as a reference). When analyzing the effects of a CXCR4 inhibitor, we found that AMD3465 treatment decreased the migration of *CALM-AF10* translocated leukemias. Following a 4hr treatment with 1uM AMD3465, U937 cells treated with 100ng/mL of CXCL12 for 24 hours had a 6-fold decrease in migration compared to cells exposed to vehicle control (**Figure 1G**, n=3, p< 0.01). Dose and time response curves with and without CXCL12 as a chemoattractant reveal migration of U937 cells over time (normalized to the 0 AMD3465 condition) is minimally impacted by increasing concentrations of AMD3465 in the absence of CXCL12. There is a trend toward diminished migration with low doses of AMD3465 in the presence of CXCL12 (**Figure 1H**). Time course experiments reveal a trend toward a greater impact of CXCL12 with short exposures to chemoattractant (**Figure 1I**: 0uM AMD3465 at 4 hour *vs* 24 hour, p=0.06). These observations indicate that AMD3465 exerts an inhibitory effect on migration of *CALM-AF10* expressing leukemia cells.

### 3.2 Increased Expression of CXCR4 in CALM-AF10 Leukemia

**Figure 2:**
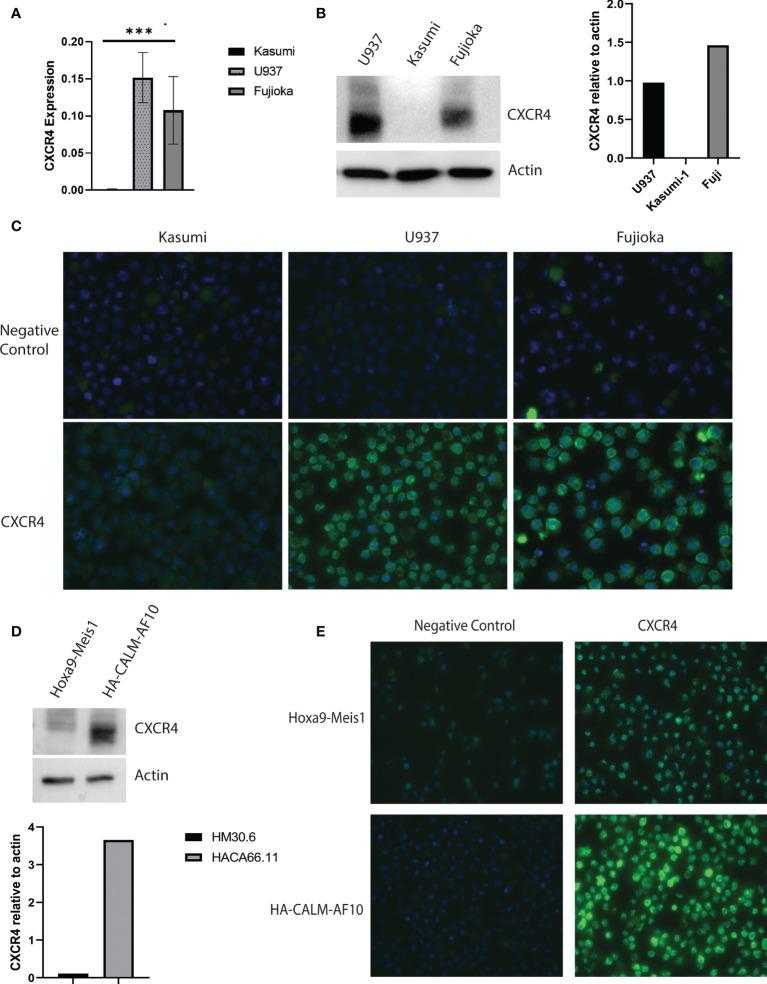
Increased CXCR4 expression in *CALM-AF10* transformed leukemia cells. CXCR4 expression is evaluated in human myeloid leukemia cell lines characterized by the *CALM-AF10* translocation (U937 and Fujioka) and compared with a myeloid leukemia cell line not containing the *CALM-AF10* translocation. Expression is examined by transcript quantification, with values shown relative to *GAPDH* **(A)**, and protein evaluation by Western blot, with corresponding densitometry normalized to actin **(B)**. Immunofluorescence staining of human leukemia cells reveals increased CXCR4 expression is concentrated on the plasma membrane **(C)**. **(D)** High CXCR4 protein expression is confirmed by Western blot in a murine leukemia induced by *CALM-AF10*, compared with a murine leukemia characterized by the *Hoxa9-Meis*1 translocation, with corresponding densitometry shown, normalized to actin. **(E)** Immunofluorescence staining in the *CALM-AF10* translocated murine leukemia cells reveals increased CXCR4 expression is concentrated on the plasma membrane. ***p < 0.001.

We assessed CXCR4 expression in U937 and Fujioka, two myeloid leukemia cell lines containing the *CALM-AF10* translocation, and compared it with that in Kasumi-1, a myeloid leukemia cell line that does not express *CALM-AF10*. The mRNA expression of CXCR4 in the U937 and Fujioka cells was 100-fold greater when compared with the Kasumi-1 cells (**Figure 2A**, p< 0.001). Consistent with the elevated transcript levels, total CXCR4 protein was also increased in *CALM-AF10*-translocated cell lines (**Figure 2B**). Immunofluorescence microscopy further revealed that CXCR4 was primarily localized at the plasma membrane, to a greater degree in *CALM-AF10* translocated leukemias (**Figure 2C**).

We determined the *CXCR4* expression in secondary murine cell line models, characterized by either *CALM-AF10* or *Hoxa9-Meis1* chromosomal translocations, to further validate the relationship between CALM-AF10 and increased *CXCR4* expression in human AML cell lines. In concordance with our findings in the human cell line models, we found an increase in total protein expression and plasma membrane receptor expression in the murine *CALM-AF10* leukemia cells relative to the *Hoxa9-Meis1* translocated cells (**Figures 2D, E**).

### 3.3 Effect of CXCR4 Inhibition in Combination With Doxorubicin

**Figure 3:**
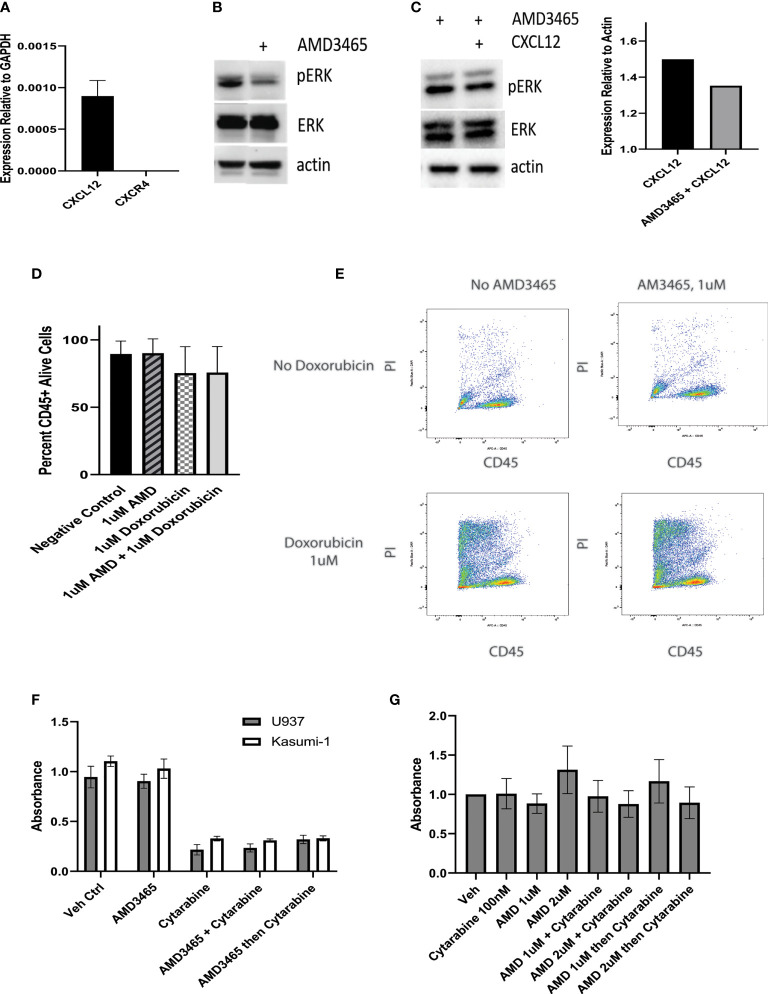
Effect of CXCR4 inhibition on cell proliferation. U937 cells are kept in co-culture with human mesenchymal stem cells (hMSCs) and the cytotoxic effect of a CXCR4 inhibitor, AMD3465 is examined. **(A)** The expression of CXCL12 and CXCR4 in hMSCs is shown, normalized to GAPDH. **(B)** AMD3465 produces a decrease in pERK/ERK in U937 cells kept in co-culture in regular media. **(C)** The impact of AMD3465 on pERK/ERK in U937 cells kept in co-culture in charcoal stripped, phenol-free media in the presence of CXCL12 is shown by western blot, with corresponding densitometry. **(D)** The cytotoxic effect of AMD3465 is examined, both with and without doxorubicin. **(E)** Representative FACS data is shown. Propidium iodide (PI) staining delineates live versus dead cells, and CD45 positivity separates the CD45+ leukemia cells from the CD45- hMSCs. **(F)** The anti-proliferative effect of CXCR4 inhibition is evaluated by BrdU assay, with and without treatment dose cytarabine. **(G)** The anti-proliferative effect of high dose and low dose CXCR4 inhibition is evaluated by BrdU assay, with and without subtoxic dose cytarabine.

We next examined the potential of CXCR4 as a therapeutic target in *CALM-AF10*-driven leukemia. We co-cultured the *CALM-AF10* expressing U937 cell line with human mesenchymal stem cells (hMSCs) to model the interaction between the bone marrow microenvironment and leukemia cells. With CD45 as a marker for leukemia cells, we employed dual color flow cytometry to separate and analyze the effect of targeted and cytotoxic treatments on leukemia cells in co-culture. We utilized this system to investigate the possible synergistic effect of AMD3465, a CXCR4 inhibitor, in combination with doxorubicin, a standard chemotherapeutic agent. We first assessed the ability of AMD3465 to inhibit CXCR4 activation by examining its effect on the MAPK pathway, a known downstream effector of CXCR4 signaling. U937 cells were cultured with hMSCs for 24hrs before treatment with 1uM of AMD3465 for 4hrs. hMSCs are known to express the ligand for CXCR4, CXCL12. We confirmed expression of CXCL12, as well as lack of CXCR4 expression, in our hMSCs *via* qPCR (**Figure 3A**). Western blot analysis revealed a decrease specifically in the phosphorylated ERK after AMD3465 treatment (**Figure 3B**), confirming an active CXCR4 signaling cascade in *CALM-AF10* expressing leukemia cells. We then examined the impact of AMD3465 on activation of the ERK pathway on U937 cells that were cultured in charcoal stripped, phenol-free RPMI and treated with CXCL12 in order to further examine the impact of specific CXCR4 inhibition. Western blot analysis again revealed a decrease in pERK/ERK in the AMD3465 treated cells (**Figure 3C**). Total ERK levels were used as a control and remained unchanged.

We next sought to evaluate the cytotoxic effect of CXCR4 inhibition. Following 16hrs of co-culture, cells were exposed to either 1uM AMD3465, 1uM doxorubicin, or the combination for 24hrs and analyzed for cell viability using flow cytometry. One micromolar dosing of doxorubicin is known to be cytotoxic to U937 cells and this was confirmed in our lab (24), and data not shown). We found that the AMD3465 treatment alone did not have a significant cytotoxic effect; the control and AMD3465 treated cells showed 89.53 percent and 90.04 percent cell viability, respectively (n=4). We also did not find a significant difference in cell viability between the AMD3465 in combination with doxorubicin (75.63 percent) and doxorubicin alone (75.33 percent) (**Figures 3D, E**).

We then investigated the potential anti-proliferative effect of CXCR4 inhibition alone or in combination with traditional chemotherapy. Cells were treated with either 1uM AMD3465, 2.5uM cytarabine, AMD3465 and cytarabine together, or AMD3465 followed by cytarabine 4 hours later. This dose of cytarabine has previously been reported to be cytotoxic to U937 cells, and we found a 77% reduction in U937 cell proliferation with 2.5uM cytarabine (25). Human leukemia cells lines U937 (*CALM-AF10*+) or Kasumi-1 (*CALM-AF10* negative) were treated for 48 hours, and cell proliferation was quantified via BrdU assay. The cytarabine treatment produced the expected anti-proliferative effect with a 70-80% decrease in absorbance in both cell lines examined. However, AMD3465 showed no anti-proliferative effect in either cell line. This was true when cells were treated with AMD3465 alone, and when AMD3465 was used in combination with cytarabine, either simultaneously or sequentially (**Figure 3F**). We then performed additional cell proliferation assays with low dose cytarabine (100nM), to evaluate whether a subtoxic dose of cytarabine would uncover a potential therapeutic effect of AMD3465. These experiments additionally incorporated AMD3465 at 2uM to assess the potential therapeutic impact of a higher dose of the CXCR4 inhibitor. We found no additive nor synergistic effect of AMD3465 at 1uM or at 2uM in combination with subtoxic doses of cytarabine, when used simultaneously or sequentially (**Figure 3G**).

## 4 Discussion

### 4.1 CALM-AF10 Transformed Cells Exhibit Enhanced Cell Migration

The *t(10;11)(p13;q14-21)* chromosomal translocation encodes the leukemogenic CALM-AF10 fusion oncoprotein. The *CALM-AF10* translocation is found in both acute myeloid and acute lymphoid leukemias and are found most frequently in T-ALL at an incidence of about 15%. *CALM-AF10* leukemia is driven by overexpression of HOX homeobox cluster genes (26, 27). The upregulation of *HOXA* genes in *CALM-AF10* leukemia is dependent on AF10’s recruitment of DOT1L, and resultant focal H3K79 hypermethylation specifically at the *HOXA* locus (8, 9). CALM-AF10 also interacts with the CRM1/XPO1 nuclear export receptor via CALM’s nuclear export signal peptide sequence, which is retained in the fusion protein, and is necessary for leukemogenesis. This interaction is required for localization of CALM-AF10 to the *HOXA* cluster, and for resultant *HOXA* overexpression (3). The PHD1-zinc-knuckle-PHD2 (PZP) domain of *AF10* is consistently interrupted in *CALM-AF10* translocations. It has been recently identified that the disruption of the PZP domain of AF10 disrupts the normal localization of DOT1L across the genome, allowing DOT1L to be tethered to the *HOXA* locus by CALM-AF10 (28).

*CALM-AF10* leukemias typically show a poor response to therapy, have an increased propensity to relapse and have a worse overall prognosis. The poor clinical outcome of *CALM-AF10*-driven leukemia necessitates the need for better understanding of how the translocation affects the behavior of the disease so that novel targeted therapies may be developed. Interestingly, *CALM-AF10* leukemias have a high prevalence of extramedullary disease, including bulky mediastinal disease, and a propensity for central nervous system relapse. Emerging evidence reveals a role for factors influencing cell adhesion and migration in these processes. While these processes may be separate from the fundamental mechanisms of tumorigenesis, they are potentially critical to tumor development, metastasis and chemotherapeutic response.

We therefore examined the migratory properties of cells transformed by *CALM-AF10* and found that MEFs transformed by *CALM-AF10* exhibited enhanced cell migration in scratch assays, compared with controls. We then examined cell migration in *CALM-AF10*+ leukemia cells via transwell migration assays and discovered that *CALM-AF10*+ U937 cells show enhanced migration in transwell assays, compared with *CALM-AF10* negative Kasumi-1 cells. Furthermore, the U937 cells showed a robust increase in cell migration toward a CXCL12 stimulus; whereas the presence of CXCL12 did not influence Kasumi-1 cell migration. Neither leukemia cell line expresses significant amounts of CXCL12, thus CXCL12 secretion and autocrine signaling are not impacting this differential response.

### 4.2 Increased CXCR4 Expression Is Seen in CALM-AF10 Leukemia and Contributes to Cell Migration

CXCL12 is the primary ligand for the chemokine receptor CXCR4, found on cell surface of HSCs and some leukemia cells. The interaction between CXCR4 on HSCs, and its ligand CXCL12, leads to HSC migration and subsequent homing to the bone marrow (29). Evidence suggests that CXCR4 plays a role in leukemia cell migration as well, and one study correlated increased CXCR4 expression on lymphoblasts with a trend toward bulky extramedullary disease in pediatric ALL (22). We investigated whether the increased cell migration we observed in *CALM-AF10* transformed cells correlated with CXCR4 expression.

Following the discovery that CALM-AF10 expressing leukemia cells migrate toward a CXCL12 stimulus, we tested whether blocking this interaction could halt migration. We found that the CXCR4 inhibitor AMD3465 abrogated this migration, confirming that CXCR4 was the integral mediator of this effect. Consistent with this finding, we found an increase in CXCR4 expression specifically in human leukemia cell lines carrying the *CALM-AF10* translocation. This finding was noted at both the transcript and protein levels. To ensure broader validity of these observations, we also confirmed our findings in murine leukemia cell lines previously generated in our lab.

CXCL12 is not only present in the bone marrow but is found in every tissue in the body, except in the CNS. CXCL12 is highly expressed in other lymphoid tissues such as the thymus, lymph nodes and spleen. It is also expressed in the liver and testes. These are all sites of potential leukemic involvement, with extramedullary involvement being characteristic of *CALM-AF10* leukemias (30). It is possible that high CXCR4 expression on the plasma membrane of *CALM-AF10* leukemias contributes to the development of extramedullary disease. Additional *in vivo* studies are needed to definitively establish the relationship between CXCR4 expression and extramedullary disease development in *CALM-AF10* leukemia.

### 4.3 The Effect of CXCR4 Inhibition on CALM-AF10 Leukemia Cells

Based on our findings of increased CXCR4 expression on *CALM-AF10* leukemic blasts, and the contribution of CXCR4 to *CALM-AF10*+ cell migration, we hypothesized that these cells would be particularly sensitive to CXCR4 inhibition as a chemosensitizer. We first established that CALM-AF10 expressing leukemia cells have an active CXCR4 signaling cascade by observing a reduction in phophoERK to ERK after CXCR4 inhibition. We then sought to examine the potential of CXCR4 inhibition to contribute to either cytostatic or cytotoxic chemotherapy effects.

Cytarabine is commonly used in the treatment of both ALL and AML. It is a pyrimidine analog and competes with cytidine for incorporation into DNA during repair and replication. When cytarabine is incorporated into DNA, it halts replication, exerting a cytostatic effect. Intriguingly, despite an intact CXCR4 signaling cascade, we found no decrease in cell proliferation upon exposure of U937 cells to AMD3465 in combination with either subtoxic or therapeutic concentrations of cytarabine.

Doxorubicin is another chemotherapy agent commonly used for the treatment of both ALL and AML. It is a cytotoxic agent, intercalating with DNA and causing double strand DNA breaks. Doxorubicin also inhibits topoisomerase II and results in apoptosis. We studied the effects of doxorubicin in combination with AMD3465 in U937 cells, and surprisingly found no synergistic nor additive effect of the combination.

Binding of CXCR4 by its ligand may result in activation of a multitude of signaling cascades, including those involved in cell survival and proliferation, such as the PI3K and ERK signaling pathways. CXCR4 inhibition is therefore an attractive target as a chemotherapeutic adjunct. CXCR4 inhibition has been examined in acute leukemia, both as a potential therapy and as a stem cell mobilizer. Previous studies have demonstrated decreased cell proliferation in AML cells *in vitro*, and decreased AML engraftment *in vivo* with the use of CXCR4 inhibitors (31, 32). A prior study reported that lymphoblastic leukemia cells in co-culture were sensitized to vincristine by a CXCR4 inhibitor through the upregulation of Bax (33). However, there are conflicting data in the published literature. For example, a 2013 study revealed that CXCL12 stimulation resulted in increased apoptosis in AML cell lines, mediated by Bcl2 family members (34). In addition, an examination of childhood ALL patient samples revealed variability in chemotactic and proliferative response to CXCR4 inhibition (35).

The factors contributing to these variable and sometimes conflicting findings are not well elucidated. CXCR4 expression and signaling is regulated through a variety of mechanisms. At a transcriptional level, CXCR4 expression may be modified through DNA methylation, directly activated by multiple transcription factors, and impacted by a range of physiologic stimuli (19, 36). Protein expression may be impacted by tyrosine sulfation or glycosylation or modified by oligomerization. There are multiple processes which separately regulate CXCR4 signaling once bound by ligand, impacting receptor internalization, degradation, and recycling (19).

Ultimately, CXCR4 expression on leukemic blasts varies, and the factors influencing differences in expression, function, and downstream signaling in leukemia are unknown. It is also not known whether degree of CXCR4 expression influences therapeutic response to CXCR4 inhibition. Herein, we identify a genetically defined a subtype of leukemia, which is characterized by high CXCR4 expression and has an intact CXCR4 signaling pathway. We report the novel findings that despite an intact CXCR4 signaling pathway, CALM-AF10 carrying leukemia cells do not show additive nor synergistic cytotoxic or anti-proliferative effects upon combinatorial inhibition of CXCR4 and traditional chemotherapeutics.

Jost et al. present a model of CNS meningeal infiltration by T-lymphoblasts through CXCR4 mediated bone marrow colonization (37). Others have provided evidence that CXCR4 inhibition prevented homing of multiple myeloma cells to the bone marrow compartment (38). It is possible the use of CXCR4 inhibitors in specific clinicopathologic scenarios may yet provide clinical benefit. The studies presented here are limited by the use of cell lines, and future studies examining CXCR4 expression and the use of CXCR4 inhibitors in patient samples will add valuable insight to the biology of *CALM-AF10* leukemia. Additional studies are needed to establish whether CXCR4 inhibition impacts other characteristics of *CALM-AF10* leukemia, including CNS invasion. We utilized a co-culture system to simulate the bone marrow microenvironment; however, additional insight would be gleaned utilizing three dimensional bone marrow scaffolds or *in vivo* models of disease (39, 40).

### 4.4 Summary

Due to its status as a G-protein coupled receptor, CXCR4 plays a role in many downstream pathways controlling cell adhesion, migration, survival and proliferation. Previous studies have reported therapeutic effect of CXCR4 inhibition in some acute leukemias in the preclinical setting, as described above. However, clinical trials of CXCR4 inhibitors have not shown promising results, particularly in ALL (41–43). Specific factors that determine response to CXCR4 inhibition have not been discovered. In this study, we demonstrate that *CALM-AF10* translocated leukemias show an increased expression of CXCR4, as well as an increase in CXCL12-stimulated cell migration. We posited that this increase would lead to an enhanced sensitivity to CXCR4 inhibitors. However, we observed no decrease in cell proliferation nor cytotoxic effect with CXCR4 inhibition. We have therefore identified a subtype of leukemia that is not sensitive to anti-proliferative or cytotoxic effect CXCR4 inhibitors, despite high levels of CXCR4 expression and an intact CXCR4 signaling pathway. This finding strongly suggests caution moving forward with CXCR4 inhibition as a therapeutic adjunct. By elucidating factors that influence response to CXCR4 inhibition, these targeted therapeutics can be better tailored to specific diseases.

## Data Availability Statement

The original contributions presented in the study are included in the article/supplementary material. Further inquiries can be directed to the corresponding author.

## Author Contributions

SF and JH performed the experiments. SF and JH wrote the paper. SZ, GS, and JS provided input into experimental design and analysis, and critically reviewed the manuscript. All authors contributed to the article and approved the submitted version.

## Funding

This work was supported by a contribution from Donna and Martin Waldron (JH). The research reported here was supported by grant U54 GM115516 from the National Institutes of Health for the Northern New England Clinical and Translational Research network (GS, JH). This work was also supported in part by the Emily M. Lyman Pediatric Leukemia Research Fund (JH), the Children’s Leukemia Research Association (JH), the Pediatric Cancer Research Fund, the Keegan Bradley Charity Golf Classic, the University of Vermont Health Network Group (JH), and the Charlotte Perelman Fund (GS). Donna and Martin Waldron were not involved in the study design, collection, analysis, interpretation of data, the writing of this article or the decision to submit it for publication.

## Conflict of Interest

The authors declare that the research was conducted in the absence of any commercial or financial relationships that could be construed as a potential conflict of interest.

## Publisher’s Note

All claims expressed in this article are solely those of the authors and do not necessarily represent those of their affiliated organizations, or those of the publisher, the editors and the reviewers. Any product that may be evaluated in this article, or claim that may be made by its manufacturer, is not guaranteed or endorsed by the publisher.
